# Unveiling the Mysteries of Non-Mendelian Heredity in Plant Breeding

**DOI:** 10.3390/plants12101956

**Published:** 2023-05-11

**Authors:** Mohsen Yoosefzadeh Najafabadi, Mohsen Hesami, Istvan Rajcan

**Affiliations:** Department of Plant Agriculture, University of Guelph, Guelph, ON N1G 2W1, Canada; myoosefz@uoguelph.ca (M.Y.N.); mhesami@uoguelph.ca (M.H.)

**Keywords:** Beavis effect, chromosomal rearrangements, cytoplasmic inheritance, epigenetics, hybridization, loss of heterozygosity, polyploidy

## Abstract

Mendelian heredity is the cornerstone of plant breeding and has been used to develop new varieties of plants since the 19th century. However, there are several breeding cases, such as cytoplasmic inheritance, methylation, epigenetics, hybrid vigor, and loss of heterozygosity (LOH), where Mendelian heredity is not applicable, known as non-Mendelian heredity. This type of inheritance can be influenced by several factors besides the genetic architecture of the plant and its breeding potential. Therefore, exploring various non-Mendelian heredity mechanisms, their prevalence in plants, and the implications for plant breeding is of paramount importance to accelerate the pace of crop improvement. In this review, we examine the current understanding of non-Mendelian heredity in plants, including the mechanisms, inheritance patterns, and applications in plant breeding, provide an overview of the various forms of non-Mendelian inheritance (including epigenetic inheritance, cytoplasmic inheritance, hybrid vigor, and LOH), explore insight into the implications of non-Mendelian heredity in plant breeding, and the potential it holds for future research.

## 1. Introduction

The field of plant breeding and genetics has traditionally been based on the work of Gregor Mendel, who first proposed the ‘laws of inheritance’ [1]. The Mendelian laws of inheritance, also known as Mendelian genetics, state that the inheritance of traits in an offspring is determined by the combination of discrete units of heredity, called genes, which are passed on from parent to offspring [2]. Mendelian genetics is based on the idea that each gene is passed on in a certain predictable way and can be used to explain the inheritance of different traits, which can be supported by three laws of inheritance proposed by Mendel: the Law of Segregation, Law of Dominance, and Law of Independent Assortment [2,3].

The Law of Dominance states that when two versions of a gene (alleles) are present, one allele will be expressed, while the other allele will be “masked” or not expressed [4,5], which is known as the dominant and recessive alleles, respectively. The Law of Segregation states that during the formation of gametes, the two copies of each gene separate so that each gamete only contains one copy of the gene [6,7]. This means that the offspring will receive only one copy of the gene from each parent, thus leading to the phenotype of the offspring being determined by the combination of the alleles from both parents [7]. The Law of Independent Assortment states that the alleles at different loci segregate independently of each other, meaning that the inheritance of one allele at a locus does not influence the inheritance of the alleles at other loci [6,7].

The Mendelian laws of inheritance can provide sufficient explanations for Monogenic inheritance (a single gene determining a single trait) traits such as seed shape, flower color, and seed coat color [7,8]. However, there are multiple examples that Mendelian laws of inheritance are not applicable for polygenic inheritance (multiple genes at different loci are involved in determining a single trait) traits such as yield, maturity, and abiotic/biotic stresses [9,10]. Those traits that are not explained by Mendel’s rules, are referred to as non-Mendelian caused by non-Mendelian heredity [8,10]. Non-Mendelian traits may be transmitted from one generation to the next in a number of different ways, such as through the action of gene-environment and/or gene–gene interactions, or even through epigenetic changes [8,9].

Gene-environment interaction is one of the most important factors in plant breeding areas when the expression of a gene is influenced by the environment [11,12]. In plant breeding, environmental factors can play a role in the expression of complex traits such as yield [13]. For example, a plant grown in a hot and dry climate may have a different yield than the same plant grown in a cooler climate [14]. Gene–gene interaction is a phenomenon in which two or more genes interact with each other to produce a phenotype that is different from the effect of either gene alone [11]. In plant breeding, non-Mendelian traits can be transmitted through gene–gene interactions, either through epistatic (complex) interactions between genes, or through the action of regulators of gene expression [8,9,11]. In addition, gene–gene interactions can play a role in the control of gene expression, allowing the expression of non-Mendelian traits to be modulated [11]. Epigenetics, which deals with the regulation of gene expression without changing the underlying DNA sequence, can affect the expression of traits, and may be inherited in the same way as non-Mendelian traits [11,15]. This means that the study of non-Mendelian heredity in plants is closely intertwined with the study of epigenetics [15,16].

Non-Mendelian traits can be used in plant breeding to create novel varieties with desired characteristics. The identification of these traits and the ability to generate novel varieties through the manipulation of these traits is a major focus of most research in plant breeding [16,17,18]. In this review, we will begin by addressing the historical context in which non-Mendelian heredity was first discovered, followed by an overview of the different types of non-Mendelian heredity and how they are manifested in plants. We will then discuss the methods used to study non-Mendelian heredity in plants, such as molecular markers and genetic mapping. Finally, we will explore the various applications of non-Mendelian heredity in plant breeding, such as its role in crop improvement and disease resistance.

## 2. The Basis of Non-Mendelian Heredity

Non-Mendelian heredity is a term used to describe inheritance patterns that do not fit the classical Mendelian inheritance model, which allows breeders to create new varieties of plants with desired characteristics in a different way [2,3,6]. By understanding the various forms of non-Mendelian heredity, breeders can better control the genetics of the plants they are working with and create varieties that are more likely to be successful [6,10]. The lack of clear-cut Mendelian inheritance patterns in plants is largely because many plants are capable of producing offspring through different reproductive strategies such as cross-pollination, self-pollination, and asexual reproduction [6,8,9,10]. This complexity results in multiple patterns of inheritance that do not fit the traditional Mendelian model.

Cross-pollination can result in offspring with characteristics that are a combination of the parental forms. This is known as hybridization and can be used to create new varieties of plants with desired traits [19,20]. However, the offspring may not always be identical to the parental forms, as the genetic material from both parents can mix and recombine (genetic recombination) in unpredictable ways [21]. Understanding the implications of non-Mendelian heredity is also important, as it can lead to a lack of genetic diversity and the appearance of harmful recessive traits [21]. Self-pollination is when a plant produces pollen and fertilizes itself, mostly resulting in offspring genetically identical to the parent [22]. While self-pollination can be used to maintain desirable traits in a species, it can also lead to inbreeding, which can reduce genetic diversity and lead to the appearance of recessive traits [23]. Asexual reproduction is another form of non-Mendelian heredity [24]. This is when a single parent reproduces by cloning itself, resulting in offspring that are genetically identical to the parent. Asexual reproduction is often used to propagate desirable traits in plants, as it allows for a greater degree of control over the genetic makeup of the offspring [24,25]. However, it can also lead to a lack of genetic diversity by reducing genetic recombination rate), which can be detrimental to the species in the long run.

Using genetic information generated by different methods such as pedigree analysis, DNA sequence analysis, linkage analysis, and genetic mapping are some of the common ways to calculate the genetic recombination rate in plants [26]. Genetic information is arranged in chromosomes of various lengths, resulting in the genetic linkage of genes [27]. For example, in green pea (*Pisum sativum*) as the main crop Mendel worked on, there are seven chromosomes, of which two and three of the genes encoding Mendel’s traits are located on chromosomes 1 or 4, respectively [28]. It is remarkable that most trait combinations indicated unlinked genes, which is explained by the large size of the pea chromosomes and the distance of the loci, leading to a high recombination frequency and no linkage disequilibrium [29,30]. If the genes were more closely linked, it would have made it difficult to observe and interpret new combinations as the result of independent segregation [30]. Mendel was able to accurately characterize the material he had available and avoided the issue of polygenic traits determined by multiple genes (quantitative inheritance) [28].

## 3. Polyploidy

Non-Mendelian heredity can also be observed in plants through polyploidy [20]. This is when a species has more than two sets of chromosomes, resulting in offspring that is genetically distinct from their parents [20]. Autopolyploidy and allopolyploidy are two of the most common ploidy in plants [31]. Autopolyploidy occurs when an individual has multiple sets of chromosomes from the same species, whereas allopolyploidy is when the individual has multiple sets of chromosomes from different species [31,32]. Some researchers distinguish between two types of polyploidy based on their origins (parentage), while others focus on genetic characteristics such as chromosomal profile and behavior [33]. Polysomic polyploids are formed when duplicated chromosomes are completely homologous and result from multivalent or random bivalent segregation during meiosis [34]. Disomic polyploids occur when duplicated chromosomes are partially homologous and strictly from bivalent homologous chromosomes [35]. Despite differences in origin, both types of polyploids have high levels of gene duplication and heterozygosity, with autopolyploids having higher levels of heterozygosity than diploids due to outcrossing [34,35]. This phenomenon can be used to create new varieties of plants with desired traits such as increased size, higher yields, or higher disease resistance, but it can also lead to reduced fertility, as the offspring may not be able to produce viable gametes [34]. Moreover, polyploid plants are reported to have slower growth rates and flowering time over a longer period than diploid plants, a trait that is advantageous for ornamental breeding [36]. Another polyploidy breeding example is the developing seedless watermelon by introducing one chromosome to its genome (triploid) [37].

### 3.1. Autopolyploidy and Allopolyploidy

**Figure 1:**
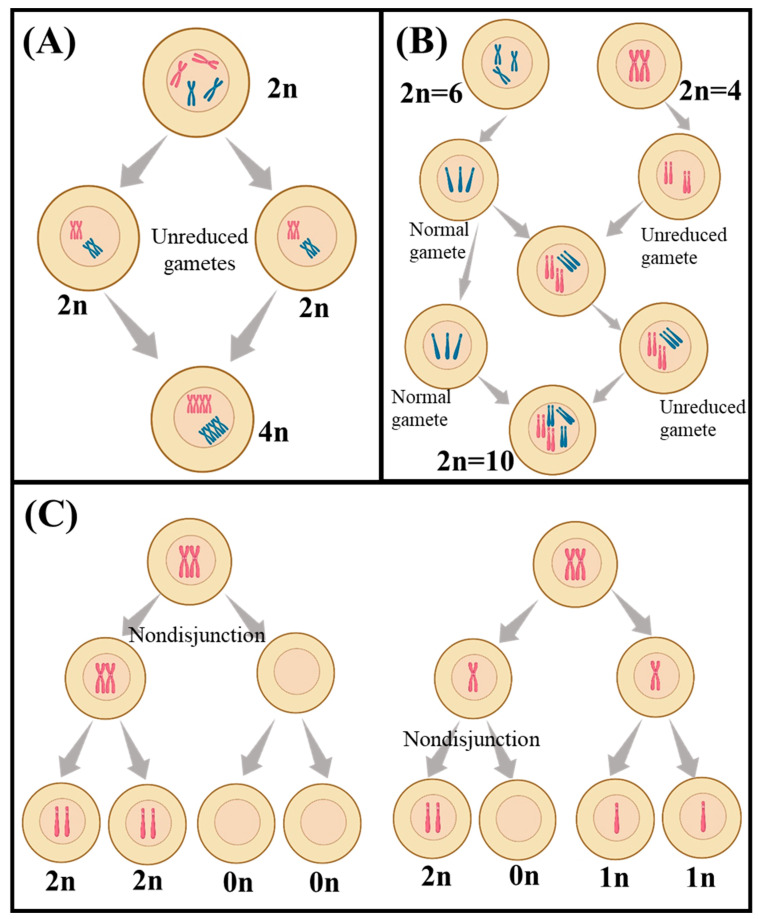
A schematic view of different polyploidization including (**A**) autopolyploidy, (**B**) allopolyploidy, and (**C**) aneuploidy. The schematic was created by using BioRender.com.

**Figure 2:**
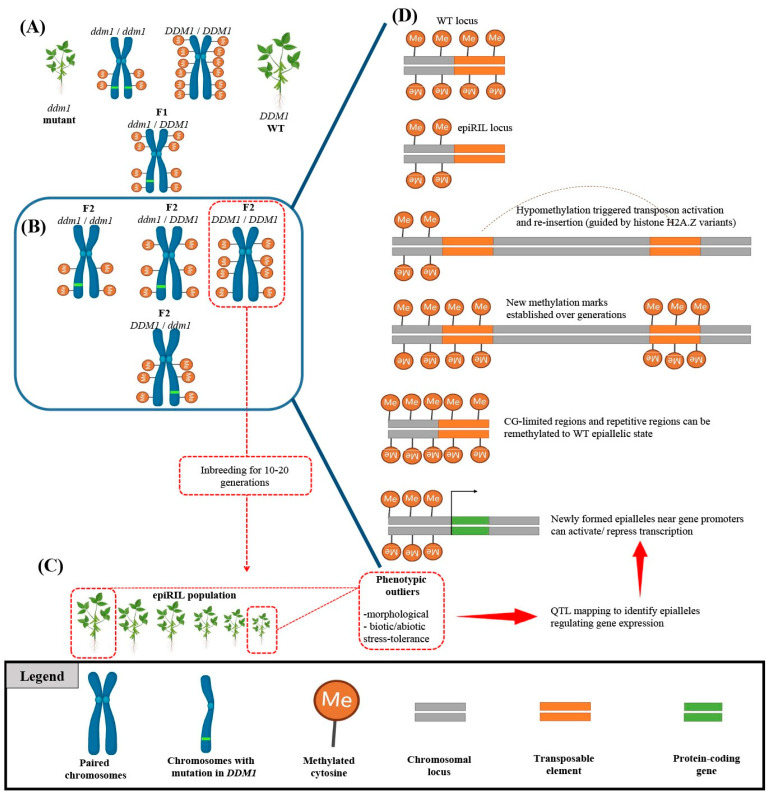
Identification of novel epialleles using epigenetic recombinant inbred line (epiRIL) generation. (**A**) Crossing wild-type (WT) plants with DNA methylation-deficient mutants such as ddm1 mutant can redistribute genome-wide methylation patterns. (**B**) Progeny carrying WT alleles are selected for multigenerational inbreeding to generate epigenetic recombinant inbred lines (epiRILs). (**C**) The epiRIL population is evaluated for variations in stress resistance or morphological traits to identify phenotypic outliers. (**D**) The identified epiRIL lines undergo an epigenetic quantitative trait loci (epiQTL) analysis to discover novel epialleles. Such epialleles can occur due to the activation of transposable elements (TEs) and their reinsertion into distant loci, determined by chromatin properties and the nature of the target sequence (CG content). The scheme was adapted from Srikant and Tri Wibowo [115] and was created by using BioRender.com.

Meiosis in polyploids is more complex than in diploids, but the expected segregation ratios can still be calculated. In autotetraploid species (Figure 1A), such as the potato, which has four sets of chromosomes from the same species, a recessive trait would be expressed in only one out of every 36 F_2_ plants [38]. This frequency could be further reduced in autohexaploid plants, with six copies of each chromosome, to one in 64 [38]. Additionally, autopolyploid plants have the potential for double reduction, which is when two recombined chromosomes move to the same pole in anaphase I, a process not seen in diploids [39]. The fact that recessive traits are expressed at different frequencies in autopolyploid and allopolyploid species (Figure 2B) is important for breeders and researchers, which allows them to predict how often are certain traits expressed in a given generation, which can help inform breeding strategies and help identify desirable traits [40,41].

### 3.2. Aneupolyploidy

Aneuploidy refers to the condition where the number of chromosomes in a cell is not an exact multiple of the haploid number of the species [42] (Figure 1C). Aneuploidy can occur naturally in plants, but it can also be induced by breeding or through the use of chemical or radiation treatments [43]. Aneuploids are often characterized by reduced fertility and can be used to generate new plants with desirable traits, as well as to study the effects of chromosome changes on plant development and physiology [42,43]. In aneuploids, random segregation leads to distorted ratios, which can result in modified trait expression [30]. In wheat, aneuploidy can lead to reduced seed size, altered flowering times, and changes in the size and shape of the leaves [30,44].

### 3.3. Heterosomes and B-Chromosomes

Heterosomes, which are sex chromosomes in dioecious plants, can also be responsible for the expression of different traits, in addition to the type of flower (sex) [45]. One example is the *Silene latifolia*, a species of flowering plant in the Caryophyllaceae family, which exhibits differences in its flower morphology based on the heterosomes it expresses [45]. The female plants have larger petals and sepals and more white flowers than the male plants, which have smaller petals and sepals and fewer white flowers [45]. Additionally, the female plants have larger leaves, while the male plants have proportionally smaller leaves [45]. This difference in morphological characteristics is due to the presence of different heterosomes in the male and female plants. In plant breeding, heterosomes are used to create novel varieties of plants with desirable traits, such as increased disease resistance, improved yield, and improved flavor [30].

B chromosomes, which exist in addition to the autosomes, can contribute to trait formation, and their asymmetric segregation patterns can lead to irregular segregation in a similar way to heterosomes [46]. Hugo de Vries, who noticed the importance of Mendel’s experiments in the early 1900s, thought that the frequent changes in the form of the evening primrose *Oenothera lamarckiana* between generations might signify the formation of new species due to mutations [47]. It was later discovered, however, that the variations were caused by the presence of extra chromosomes or an imbalance in the amount of a certain element, not by changes in the order of DNA sequence [47]. Jones and Ruban [48] reported the effectiveness of the B chromosome in accelerating crop improvement, particularly in grasses. However, to what extent and how much B chromosomes can contribute to trait formation is still not well explored. In the case of *P. sativum*, there are no sex or B chromosomes, however, some crosses have yielded trisomic pea plants with three copies of an individual chromosome rather than the expected two, creating significant differences in the phenotype [49].

Overall, polyploidy is a useful tool for creating new varieties of plants with desired traits, but it also has the potential to lead to reduced fertility in the offspring. Therefore, careful consideration needs to be given when using this technique to ensure that the desired results are achieved without sacrificing fertility.

## 4. Cytoplasmic Inheritance

Cytoplasmic inheritance is a type of non-Mendelian inheritance that occurs in plants, animals, and fungi. In nuclear inheritance, genetic information is passed from one generation to the next through the DNA found in the nucleus [50]. In cytoplasmic inheritance, genetic information is passed on from one generation to the next through the cytoplasm, which contains many different types of organelles and other cellular components, including mitochondria and chloroplasts [50,51]. There are several common examples of cytoplasmic inheritance, such as cytoplasmic male sterility (CMS), mitochondrial mutations, and chloroplast inheritances, especially in plant breeding areas [51].

### 4.1. Cytoplasmic Male Sterility (CMS)

CMS is considered as a type of non-Mendelian inheritance because it is inherited solely through the cytoplasm of the female parent and is not determined by the genes of either parent, in which the male reproductive organs of the plant are non-functional [52,53]. It is a form of genetic male sterility and is used extensively in plant breeding programs to produce hybrid varieties [53,54]. The trait is caused by a mutation in the mitochondrial genome which results in the production of aberrant proteins that interfere with the normal function of the male reproductive organs. CMS is used to produce hybrid varieties of crop plants by crossing a sterile CMS variety with a fertile restorer variety [54,55]. The hybrid progenies are all fertile, and this hybrid vigor results in increased yields and improved disease resistance.

CMS can be used to produce homozygous lines for specific traits, which eliminates the need for tedious hand-pollination for hybrid seed production [55]. However, the pollen from male sterile lines is often of poor quality, which requires specific conditions for storing [53,55]. In addition, CMS may cause a decrease in genetic diversity, as most commercial hybrids are produced by crossing two inbred lines and lead to a decrease in outcrossing, as the male sterile lines are not able to produce viable pollen [54].

### 4.2. Mitochondrial Inheritance

In recent years, mitochondrial inheritance has been used increasingly in plant breeding to create improved varieties of crops with higher yields, improved disease resistance, and improved nutritional value [56]. By introducing specific gene mutations into the plant’s mitochondria, breeders can control the expression and activity of certain genes and alter the plant’s phenotype. Plant mitochondrial genomes are larger and less conserved than chloroplast genomes, therefore, have received less attention [57]. In a study conducted by Forner et al. [58], transcription activator-like effector nuclease (TALEN)—gene-drive mutagenesis (GDM) was introduced to mutate tobacco mitochondrial *Nad*9 gene, resulting in a collection of mutants that have a single amino acid substitution in the Nad9 protein. These mutants are homochondriomic and can be stably inherited in the expected maternal fashion [58].

The use of mitochondrial mutations in plant breeding has been gaining attention in recent years as a tool for improving a variety of plant traits. Rauf [59] reported the use of mitochondrial mutations to create a drought-tolerant sunflower, resulting in an increase in the amount of unsaturated fatty acids in the plant’s seed oil, which increased the sunflower’s tolerance to drought stress. Similarly, mitochondrial mutations were used to increase the yield of rice plants by increasing the amount of photosynthetic efficiency [60]. These mutations also increased the plant’s tolerance to heat stress [60]. Mitochondrial mutations were successfully applied to change the flower color of petunia with a mutation in the mitochondrial gene’s coding region [61].

### 4.3. Chloroplast Inheritance

Chloroplast inheritance in plant breeding refers to the transmission of chloroplast DNA from the female parent to all of the progeny of a cross [62]. Chloroplasts are organelles within the cells of plants that contain their own genetic material, known as plastomes. These plastomes can be inherited from a female parent, allowing breeders to track the maternal parent in a cross, and can be used to develop male-sterile lines [62,63]. Chloroplast genomes in plants are highly conserved sequences of 100–150 Kb containing around 100 genes [64]. The standard structure of a chloroplast is composed of four elements, including inverted repeats that divide the large and small single-copy regions [65]. They have been a popular choice for plant identification due to their high copy numbers in the cell [66]. Previous methods of chloroplast isolation or PCR amplification were challenged by the same sequences existing in both the nuclear and mitochondrial genomes [67,68,69]. Recent techniques have made it easier to determine the accurate sequence of the chloroplast by taking advantage of its higher abundance in short-read sequencing [67,68,69].

Analysis of polymorphisms in 2580 soybean accessions, including 107 wild soybeans, revealed that the chloroplast genome is more variable than the mitochondrial genome in terms of variant density [70]. Cultivated soybeans harbored 44 chloroplast haplotypes and 30 mitochondrial haplotypes, with the two most frequent types accounting for nearly 70 and 18%, respectively [70]. Wild soybeans, on the other hand, had 32 chloroplast and 19 mitochondrial haplotypes. However, only a small proportion of cultivated soybeans shared cytoplasm with wild soybeans. Two mitochondrial polymorphism sites were discovered to be heterozygous in most soybeans, suggesting a link between heterozygosity and domestication, improvement of landraces, geographic adaptation [70]. The haplotypes of many soybean cultivars could be beneficial for evaluating the impact of cytoplasm on performance, as well as for breeding cultivars with desired cytoplasm. It is possible that mitochondrial heterozygosity is associated with soybean adaptation, which requires further investigation [70].

Maternal inheritance of cytoplasmic organelles (mitochondria and chloroplast) predominates in eukaryotes, thereby, preventing the organellar genomes from being recombined through sexual reproduction. However, any mechanisms underlying materials’ heredity are not well understood. Chung et al. [11] reported the effect of environmental conditions on the maternal inheritance of cytoplasmic organelles. Mild chilling stress during male gametogenesis can lead to paternal plastid entry into sperm cells and significantly increased paternal plastid transmission [11]. This research has revealed that paternal plastid inheritance is controlled by a gene-degrading exonuclease in mature pollen [11]. In certain environmental conditions, maternal inheritance can be disrupted, caused by a combination of an organism-blocking mechanism and a gene-destroying system. Ultimately, the inheritance of plastids is determined by the combination of genetic and environmental factors [11].

## 5. Chromosomal Rearrangements

Chromosomal rearrangements are a type of genetic alteration that can be used in plant breeding programs to produce new varieties with desirable traits [71]. Rearrangements involve breaking and rejoining sections of chromosomes, resulting in rearrangements of gene order, deletions or additions of genetic material, or changes in chromosome structure [71,72]. These changes can lead to new combinations of genes that can confer desirable traits. Chromosomal rearrangements were successfully implemented in different breeding programs to increase the genetic variation of wheat [73], maize [74], and rice [75]. Zhang et al. [75] examined the effects of multiple DNA double-strand breaks (DSBs) in rice plants and found that rice varieties with a high number of simultaneous DSBs (e.g., over 50) showed low-frequency large chromosomal deletions and duplications, but this was not the case for plants with lower order DSBs (e.g., under 10). Therefore, large chromosomal rearrangement can occur in varieties with a large number of DSBs [75]. In another study conducted by Sharma and Peterson [74], it became demonstrated how transposon-induced chromosomal rearrangements can rapidly and progressively increase genetic variation and have a major impact on genome evolution in maize.

In addition, chromosomal rearrangements can be used to develop biotic stress resilience crops in plant breeding programs. In order to understand the Ty-1 locus—a resistance gene for tomato yellow leaf curl virus (TYLCV), which is found in *Solanum chilense* and has been used in breeding for TYLCV resistance—research was conducted using 19 markers from tomato chromosome 6 in two commercial hybrids [76]. Fluorescence in situ hybridization (FISH) revealed two chromosomal rearrangements, and 30 recombinants were identified between *Solanum lycopersicum* and *Solanum chilense* in the Ty-1 introgression [76]. The results of this study provided useful information for future tomato breeding programs in terms of selecting the resistance line accurately in a timely manner [76].

## 6. Gene–Gene Interaction

Gregor Mendel conducted dihybrid crosses to examine how genes can affect traits. In Gregor Mendel’s experiments, he crossed a homozygous plant with round and yellow seeds (RRYY) with another homozygous plant with wrinkled and green seeds (rryy) and observed a phenotypic ratio of 9:3:3:1, where each gene locus had an independent effect on a single phenotype [6]. Nevertheless, in numerous instances, complex phenotypes do not adhere to the principles of segregation and independent assortment elucidated by Mendelian genetics, as they are frequently governed by the contribution of multiple genes to their ultimate expression [77]. When two genes contribute to the same phenotype (gene–gene interaction), the phenotypic ratio may deviate from that expected from the independent action of each gene, a phenomenon known as epistasis [78]. Such interactions between two or more loci can create novel phenotypes for which the allelic effects of single genes are described as “dominant” and “recessive” [78]. Epistasis is a phenotypic-level phenomenon, wherein an independent assortment of genotypes is observed, yet the phenotypic outcomes may differ from the anticipated ratios [78].

Shull [79] seminal study of the weedy plant Bursa bursa-pastoris, more commonly known as Shepard’s Purse, is a classic example of epistasis. Upon crossbreeding doubly heterozygous plants, Shull observed a ratio of 15:1 between triangular and oval capsules, respectively [79]. This phenomenon is thought to be the result of two pathways, each containing a dominant locus that produces the triangular shape [79]. When both pathways are blocked by recessive alleles, an oval-shaped seed capsule is produced, a phenomenon known as recessive-by-recessive interaction [79]. This suggests that having two recessive genotypes results in a different phenotype than having just one from either locus. Recent decades have seen a major breakthrough in the field of genome-wide studies, which typically involve single-locus analysis of variants and their correlation to a certain phenotype [80,81]. Despite this, many genetic studies of complex traits have failed to yield results due to the potential interactions between loci [82].

Epistatic interactions between quantitative traits can manifest in two forms: a change in the magnitude of the effects or a change in the direction of the effects [78]. In the absence of epistasis, the estimates of the additive and dominance effects at each locus remain the same regardless of the genotype of the other locus [78,83]. However, with epistasis, the effect of one locus depends on the genotype at its interacting locus [83]. There is still much debate about the relevance of epistasis to quantitative traits, with some concentrating on individual genotypes and others focusing on the epistatic genetic variance in populations [83]. Genetical epistasis is independent of allele frequencies, whereas the total genetic variance in a population is divided between additive, dominance, and epistatic variance, which are all based on allele frequencies [84]. Epistasis can cause different effects in populations because the effect of one locus is dependent on the allele frequency of another locus. Its influence can be strong in one population and weak or even reversed in another [84]. A lot of additive genetic variance is produced when both loci are at intermediate frequencies. Unless the genotypic values of one locus are opposite in different contexts, the additive genetic variance is usually the main source of total genetic variance for a range of allele frequencies when epistasis is present [83].

Most genetic variance that is observed for quantitative traits is additive, which could be either ‘real’ or ‘apparent’ due to epistatic gene action at many loci [85]. This is significant for the purposes of heritability and predicting phenotypes; however, it is especially important when trying to understand the effects of genetic drift and inbreeding, as well as the genotype-phenotype map, long-term responses to selection, and genetic interactions [85]. In order to differentiate between ‘real’ and ‘apparent’ additive genetic variance, we must investigate the presence of epistasis and calculate the genotypic values at related loci that could be involved in epistatic activities or other higher-order interactions [78,85]. Model plants allow us to determine epistatic interactions through mutations developed in the same homozygous genetic background, quantitative analysis of inbred and outbred populations, chromosome substitution, introgression and near-isogenic lines, and induced mutations [78,85]. The ability to construct mapping populations from crosses of inbred lines with allele frequencies of 0.5 is notably advantageous since it increases epistatic variance and the prevalence of two-locus genotypes [78].

Epistasis can be studied through the examination of mutants in the same homozygous genetic background [86]. Epistasis occurs if the difference in phenotype between the double mutant cannot be predicted by the combined effects of the two single mutants [87]. This can either be negative or synergistic, meaning that the double mutant is more mutant than expected, or positive, meaning that the double mutant is less mutant than expected [86,87]. This method is advantageous as the interacting partners are known, allowing the construction of genetic interaction networks. However, it is difficult to scale this method to large numbers of mutations, as it requires the generation of almost ~n^2^ genotypes to thoroughly explore the interaction space [86].

The extent to which the intricate epistasis indicated by induced mutation studies applies to natural populations can be examined through the use of inbred lines, artificial selection lines, chromosome substitution lines, and the mapping of quantitative trait loci (QTL) associated with complex traits via linkage and association mapping [88]. Linkage mapping is done by breeding two lines that differ in the trait of interest and measuring the genotypes and phenotypes of the mapping population [89]. Association mapping uses samples of individuals or inbred lines from a natural or unrelated population and looks for a significant difference in phenotype between marker genotypes [90]. Association mapping has the ability to capture more genetic diversity and has increased precision but is prone to artefactual linkages caused by population structure and has reduced power to detect QTL with minor allele frequencies below 0.5 [78,90].

In QTL mapping, epistasis can be estimated by a statistical model with factors for each QTL and the interaction between them [78]. Multifactorial perturbations can be used to screen for epistasis with a small number of individuals, which is more efficient than constructing all possible gene combinations [78]. Power to detect epistasis is highest in inbred lines due to the equal frequencies of each allele [91]. However, in small mapping populations, the number of individuals with rare homozygous genotypes is small, which increases the variance of the phenotype [78]. Additionally, other loci can produce confounding effects, and multiple testing can make it difficult to detect epistasis. Most studies only assess additive effects, but epistatic effects can be as large as main effects and can occur between non-significant loci [91,92]. Epistatic interactions have been observed in genetic studies of growth rate and metabolites in *A. thaliana* [93] and differences in inflorescence and whole-plant architecture in maize and teosinte [94]. These findings demonstrate that epistasis must be considered in order to understand the genetics of complex traits. However, QTL mapping cannot be used to pinpoint the particular genes involved in the interaction because the QTL intervals contain many genes. Using model organisms, it is possible to further dissect QTL [95]. Near-isogenic lines can be created in which a QTL region is incorporated into a single genetic background, and then successive generations of recombination are used to isolate the exact genomic interval. *A. thaliana* near-isogenic lines were used to show two epistatically interacting QTLs, which had opposite effects on growth rate depending on the genetic context [95]. Transformation and allelic replacement can be used to prove variants are causal and to construct all possible combinations of variants to investigate epistasis at the nucleotide level [95].

By introgressing fragments of DNA from one genotype into the genetic background of another, it is possible to create a powerful QTL mapping design [96]. This can be done either by introducing entire chromosomes or with smaller fragments across the genome. While only a small number of introgression lines are necessary, they can be used to map QTLs with high accuracy [96,97]. Epistasis occurs when the combined effect of the introgressed fragments is not the same as the average difference in phenotype between the two parental strains [78,97]. Epistatic interactions between loci can lead to distinct main effects of each locus, as well as a failure to replicate estimated QTL effects when allele frequencies between populations vary [96]. To examine this, model organisms can be used to construct mapping populations with variable QTL allele frequencies, and the resulting in the lack of replication of QTL effects can indicate the presence of interacting loci [96].

Analysis of interactions between induced mutations is advantageous in that the participants are precisely identified. However, it is not suitable for large numbers of mutations [98]. Analysis of epistasis between QTL can evaluate interactions between numerous polymorphisms and genes, yet there is a high risk of false-positive associations due to the multiple testing penalty [98]. An alternative technique is to perform single-dimensional screens, which evaluate the phenotypic effects of a known mutation in different genetic backgrounds, though this has yet to occur on a considerable scale. Waddington [99] observed the discrepancy between the large effects of mutations and their phenotypic variability, in addition to the consistent observance of the wild-type genotype despite the presence of environmental and genetic disturbances. He coined the term ‘canalization’ to describe the process of suppressing the effects of variation in response to these perturbations. In modern terms, genetic canalization involves the dampening (less than additive) relationship between genetic variants that segregate naturally [99]. By examining the modification of a mutant allele’s effects by these naturally segregating variants, one can gain insight into the nature and strength of the naturally occurring epistatic modifier loci [99].

Crossing a mutant allele to a sample of wild-derived lines and assessing the F1 genotypes’ phenotypes is a variant of the mutant introgression design [78]. This is simpler to implement than constructing introgression lines, however, it cannot attribute any phenotypic variation to allelic or non-allelic complementation without using a QTL-mapping population [78]. An experimental design can be adapted to evaluate the effects of naturally segregating epistatic modifiers of mutations that affect quantitative traits in natural populations. This includes assessing the additive effects of the mutant and wild-type alleles of the locus in question in different genetic backgrounds, either as an introgression or an F_1_ design [100]. A significant interaction between the mutant and background genotypes would indicate the presence of epistasis. Examples include maize’s hypersensitive response with the Rp1-D21 disease resistance mutation and *Arabidopsis thaliana*’s morphological and life history traits with a heat shock protein (HSP)90 RNAi knockdown allele [101,102].

## 7. Epigenetics

Epigenetic modifications (e.g., DNA methylation, chromatin remodeling, histone modification, and RNA-directed DNA methylation) can be defined as any changes in gene expression without alterations in the DNA sequence [103]. It is well-documented that epigenetics plays a fundamental role in plant growth and development by regulating gene expression [103,104,105,106,107]. Furthermore, the discovery of epialleles, which can be defined as genetic variations caused by changes in DNA methylation, has opened up a new understanding of how epigenetic modifications can lead to novel phenotypes and contribute to evolution [108]. Since epigenetic modifications can be influenced by a range of environmental stressors, the epigenetic state of an individual can be highly plastic and can be influenced by both internal and external factors, leading to epialleles being transmitted to offspring without following traditional Mendelian inheritance patterns [109,110,111]. This non-Mendelian behavior of epialleles has important implications for understanding and studying inheritance, as well as for the fields of evolution and ecology [111,112,113]. It also has practical implications for plant breeding, where understanding the non-Mendelian inheritance of epialleles can help to develop crops that are better adapted to changing environmental conditions and exhibit improved yield potential through epigenetic recombinant inbred lines (epiRILs) (Figure 2) [113,114]. The use of epiRILs allows breeders to employ the function of epigenetic modifications in gene expression and phenotypic variation in a controlled genetic background, reducing the confounding effects of genetic variation [107,115].

The creation of epiRILs involves several steps. First, two parents with different epigenetic states are crossed to produce an F1 generation. The F1 plants are then selfed for multiple generations to create a genetically homogeneous population with different epigenetic states [107]. The resulting epiRILs are then genotyped and phenotyped to identify genetic and epigenetic factors that contribute to phenotypic variation. Indeed, by genotyping and phenotyping epiRILs, it can be identified regions of the genome that are associated with phenotypic variation and epigenetic modifications [115]. This can lead to the identification of epigenetic modifications that are associated with specific phenotypes and can be used to develop crops that are better adapted to changing environmental conditions [107,116].

Several studies have used epiRILs to study the role of epigenetic modifications in gene expression and phenotypic variation [117,118,119,120,121,122,123]. For example, Zhang et al. [124] used epiRILs to study the role of DNA methylation in gene expression and drought tolerance in *Arabidopsis thaliana*. They identified several differentially methylated regions that were associated with drought tolerance, and demonstrated that DNA methylation can play a critical role in gene expression and drought tolerance [124].

In another study, Miura et al. [125] used epiRILs to study the role of histone modifications in gene expression and flowering time in rice. They found several differentially modified histone marks that were associated with flowering time, and identified that histone modifications can play a critical role in gene expression and phenotypic variation [125].

The use of epiRILs has also been applied to crop improvement. For example, Xu et al. [126] used epiRILs to study the role of DNA methylation in yield and stress tolerance in maize. Xu et al. [126] identified several differentially methylated regions that were associated with yield and stress tolerance and demonstrated that DNA methylation can be used to improve yield and stress tolerance in maize.

Although the use of epiRILs can result in getting in-depth insights of the role of epigenetic modifications in gene expression and phenotypic variation, and can be used to develop crops that are better adapted to changing environmental conditions [107], further studies using epiRILs are needed to fully understand the complex interactions between genetic and epigenetic factors in gene expression and phenotypic variation.

## 8. Gene-Environment Interaction

In most breeding programs, the inheriting genetic traits are not determined by the laws of Mendelian genetics, because of the interaction of environmental factors and the genetic makeup of the plant. Gene-environment interaction (GEI) refers to the effect of the environment on the expression of genetic variation, and it is known to play an important role in the heritability of traits in plants. Most of the complex traits that are significantly under control with environmental factors have lower heritability than others that are less affected by the environment and mostly affected by genetics.

Lower heritability traits in plants can have several disadvantages, which can have a negative impact on the pace of breeding program. One of the main disadvantages of lower heritability traits in plants is that they are more difficult to select for [127]. When selecting for traits, it is generally easier to select for high heritability traits, as they are more likely to be passed on to the next generation (i.e., follow Mendelian genetics). Low heritability traits, on the other hand, are more likely to be changed during the selection process, as they are less likely to be passed on to the next generation. Another disadvantage of lower heritability traits in plants is that they are more prone to environmental influences. Although all traits are subject to some degree of environmental influence, low heritability traits are particularly sensitive to environmental changes. This can lead to unpredictable changes in the phenotype of the plant, which can be difficult or impossible to control.

## 9. Linkage and Association Mapping in Plant Breeding

Soon after discovering Mendelian heredity, several breeders demonstrated that some traits in their crosses did not adhere to Mendel’s principles of heredity and seemed “coupled” [128]. To explain this phenomenon, scientists proposed a hypothesis that certain traits must be inherited together, e.g., through the linkage of certain genes [128,129]. This hypothesis was later verified through further experiments, which determined that certain alleles were always inherited together [128]. This phenomenon was then referred to as genetic linkage. In genetics, linkage refers to the tendency of certain genes or genetic markers to appear together more often than expected by chance [128]. Linkage occurs when two or more genes are located close to each other on the same chromosome.

If genetic linkage is prevalent in the plant genomes, why did Mendel not detect it through his experiments on pea plants? Mendel studied seven genes in pea plants, which have seven chromosomes. Although Mendel did not select gene pairs that always resided on separate chromosomes, some of the gene pairs studied by Mendel were found to be located on the same chromosome [2]. Other scientists have undertaken experiments involving the crossing of pea plants that could have demonstrated linkages: *i-a*, *v-fa*, *v-le*, and *fa-le* [29]. However, all the gene pairs, excluding one gene (*v-le*), were too far apart for Mendel to observe linkage. This implies that, although these gene pairs are syntenic, they are not statistically linked, leading to their independent assortment [29]. The *v-le* cross, however, could have shown a linkage if Mendel had conducted the experiment. It is feasible that, with one more cross, Mendel could have identified a linkage himself.

Crossing genetically different parents is the initial step in generating linkage maps and locating genes related to the desired trait. Different types of genetic populations have been formed for mapping traits, such as F_2_, F_2:3_, backcross introgression lines (BILs), recombinant inbred lines (RILs), near-isogenic lines (NILs), multiparent advanced generation intercross (MAGIC) populations, and association mapping populations based on natural populations [130]. Bulk segregant analysis (BSA), F_2_, and backcross populations are commonly used in short-term molecular mapping populations, but RILs, NILs, doubled haploid (DH), nested association mapping (NAM), and MAGIC populations, can be used for more precise phenotyping and sharing between breeders over a longer period of time [131].

Afterward, genetic linkage can be mapped using different types of molecular markers such as microsatellites, restriction fragment length polymorphism (RFLP), amplified fragment length polymorphism (AFLP), or single nucleotide polymorphisms (SNP) markers [130]. These markers allow breeders to determine the linkage between alleles at different loci on the same chromosome. Family-based linkage (QTL mapping), association mapping, and family-based association mapping are the three main linkage mapping strategies used to dissect the genetic basis of a trait of interest [131]. Family-based linkage mapping is a method of mapping genetic loci that involves analyzing the genetic data of either single or multiple families of progeny from a cross between inbred lines to identify regions of the genome that are linked to a trait of interest [132]. The resolution of this method is low due to the limited number of recombination events, but it has high power [133]. Association mapping is a technique that utilizes generations of unrelated genotypes which have experienced several recombination events over time to better locate the causal variants [134,135]. In order to increase the effectiveness of association mapping to identify rare alleles that have a major effect on the desired trait, a large sample size (thousands or more) would be essential [135,136].

Family-based linkage mapping is similar to family-based association mapping in that it involves multiple families of segregating progeny from crossing different parental lines [132]. The main difference lies in the fact that family-based association mapping assumes relatedness between the parental lines via identity by descent (IBD) and linkage disequilibrium (LD) of alleles, allowing for greater resolution of identifying variants associated with complex traits [136]. With family-based linkage mapping, however, the parental lines are assumed to be unrelated, so alternative alleles are modeled as distinct haplotypes, and this results in less resolution for QTL detection [132].

Family-based linkage mapping is frequently used with low-density genotyping technologies [137,138], while family-based association mapping necessitates a higher density of markers [139]. Family-based association mapping is not as popular in human genetics as association mapping due to the expensive recruitment of participants [140], and is also less powerful when it comes to detecting QTL due to the small amount of progeny per family. However, for plants and experimental animals, Family-based association mapping is more suitable as parents are often fully inbred lines, and large families can be created through deliberate mattings [140,141]. Since plant breeders often cross a few elite inbred lines or varieties with a range of new inbred lines or varieties to generate numerous segregating [141], Family-based association mapping can be used in existing breeding programs.

### Beavis Effect

The main aim of genetic mapping analyses is to pinpoint the genes that have major effects on the different expressions of a trait among the tested genotypes [142]. For this aim, the data gathered from a sampled population is used to estimate the true genetic impacts of QTL in explaining the phenotyping variance [142]. Several statistical methods have been used recently for QTL mapping identification to estimate the genetic effects of the population [143,144]. However, only QTL with test statistics that exceed a certain value (threshold) are commonly reported. The expected effects of the QTL are greater than actual values because they come from a cut-off distribution. Most QTL mapping procedures are capable of detecting QTLs with large effects; however, they are not as successful at identifying QTLs with intermediate and small effects [144].

Beavis and Wilkinson [145] conducted a simulation study that found that when there were only 100 progenies evaluated, the estimated phenotypic variances related to correctly identified QTL were overestimated. This became less pronounced with 500 progenies evaluated and was close to the true magnitude with 1000 progeny. This phenomenon is referred to as the Beavis effect (Winner’s Curse). When the sample size is small (e.g., 100), the statistical power of detecting a small QTL is as low as 3%, and the effects are usually exaggerated 10-fold [146,147,148].

The Beavis effect can be used to interpret the results of a meta-analysis of QTL mapping [148]. If an experiment is repeated several times, the average effect of a chromosome location are distorted if the QTL is not seen in every replicate [149]. This should be taken into account when determining if a particular marker should be incorporated into a marker-assisted selection program for a quantitative trait [149]. Several methods have been developed to alleviate the Beavis effect, however, most of them are precisely worked in a large population (*n* > 500) [150,151,152]. Beavis effects mostly affected linear approaches such as mixed linear models [150]. However, the Beavis effect can be alleviated by the recent advances in the use of machine learning algorithms in linkage and association mapping. Machine learning algorithms are not able to directly estimate the marker effects, meaning that the Beavis effect may either not be a problem, depending on how the statistical test and tunning parameters operate. However, more experiments need to be conducted to explore the possible use of advanced statistical and mathematical approaches to alleviate the Beavis effect in detecting the true genomic regions associated with the trait of interest.

## 10. Loss of Heterozygosity (LOH)

Loss of heterozygosity (LOH) is an important phenomenon in plant breeding, as it can lead to significant changes in the genetic composition of a population [153]. LOH is a type of non-Mendelian heredity that occurs when a plant loses one of its two alleles due to the mutation of a gene [153,154]. When this happens, the plant will no longer have two copies of a particular gene and will only have a single copy of that gene [154]. This, in turn, can lead to a decrease in the genetic diversity of the population, as well as a reduction in the effectiveness of selection. In plant breeding, the formation of homozygous lines through inbreeding can lead to eliminating some alleles, resulting in reduced genetic diversity. Furthermore, the outcrossing of related varieties can also lead to LOH, as the offspring will not inherit a full complement of alleles from both parents.

Loss-of-heterozygosity was reported in somatic cells of rice hybrids for the first time by Wang et al. [155], which involves the selected plant ‘AMR’, of the Chinese rice cultivar ‘ZhongxinNo.1′, as one parent. Variations were identified in the vegetative parts of the same plant using random amplified polymorphic DNA (RAPD) markers and molecular assays [155]. All F_2_ panicle rows from F_1_ hybrids involving AMR became fixed for all assayed RAPD markers, and this genotype fixation was confirmed by field observations of the F_3_ progenies [155]. The results suggested that in these hybrids, both parental homologues of some chromosomes in somatic cells are not always present. Later, Wang et al. [156] proposed a new biological mechanism called ‘assortment mitosis’, to explain this phenomenon. This mechanism can develop uniform progenies as early as the F_2_ generation and shorten the time required to obtain fixed non-parental type progenies for subsequent performance trials [156]. In another study conducted by Wang et al. [157], the root meristem cells of the rice line AMR, which causes loss of heterozygosity in its hybrids, were observed to exhibit both normal and assortment mitoses. In the case of normal mitosis, chromosomes did not form homologous pairs at metaphase, whereas in assortment mitosis, varying numbers of paired homologues were seen [157]. This suggests a mechanism for genotype fixation in rice hybrids using AMR [157].

In general, the effect of LOH on plant breeding can be profound, including reducing the genetic diversity of a population, decreasing its ability to adapt to changing environmental conditions, leading to inbreeding depression, decreasing fertility, and increasing susceptibility to disease. In addition to reducing the genetic diversity of a population, LOH can also lead to a decrease in the number of desirable traits that can be selected in a breeding program. This is because the desirable traits that were present in the parents are lost in the offspring. As a result, the available genetic material for selection is reduced, limiting the plant breeder’s ability to select desirable traits. However, LOH is a phenomenon in plant breeding that has both positive and negative effects. While most researchers focus on the potential negative effects of LOH, there are also some positive aspects that can be beneficial for a plant breeder. One of the most significant positive effects of LOH is that it can improve the uniformity of a particular variety. When a plant has a high level of heterozygosity, the outcomes of different crosses can be unpredictable, leading to a wide range of different characteristics in the offspring. By eliminating some of the genetic diversity, LOH can help ensure that the plants produced are more uniform and consistent, which is especially important when breeding plants for a specific purpose. LOH can also be beneficial for a plant breeder because it can help to speed up the breeding process, by reducing the amount of genetic diversity in the hybrid derivatives.

## 11. Conclusions

In conclusion, non-Mendelian heredity has become increasingly important in plant breeding. It can be influenced by several factors that are not related to the genetic architecture of the plant, such as epigenetics, methylation, cytoplasmic inheritance, and hybrid vigor. By exploring these non-Mendelian heredity mechanisms and their implications for plant breeding, we can better understand their prevalence in plants and how they can be used to accelerate the pace of crop improvement. This review paper provides an overview of the various forms of non-Mendelian inheritance, their mechanisms, patterns of inheritance, and applications in plant breeding. It also highlights the potential that non-Mendelian heredity holds for future research and its implications for plant breeding. By gaining a better understanding of non-Mendelian heredity, plant breeders can have more tools to develop better varieties of plants that are more resistant to disease, climate change, and other environmental factors. Ultimately, this will lead to improved yields and a more sustainable plant breeding approach.

## Data Availability

Not applicable.

## References

[1] Priyadarshan P. (2019). Plant Breeding: Classical to Modern.

[2] Allen G.E. (2003). Mendel and modern genetics: The legacy for today. Endeavour.

[3] Gautam A. (2018). MendelTs Laws. Encyclopedia of Animal Cognition and Behavior.

[4] Marks J. (2008). The construction of Mendel’s laws. Evol. Anthropol. Issues News Rev..

[5] Zhang J. (2023). What Has Genomics Taught An Evolutionary Biologist?. Genom. Proteom. Bioinform..

[6] Patwardhan D. (2022). Mendelian Principle of Inheritance. Genetics Fundamentals Notes.

[7] Mackay T.F., Anholt R.R. (2022). Gregor Mendel’s legacy in quantitative genetics. PLoS Biol..

[8] Xu S. (2022). Review of Mendelian Genetics. Quantitative Genetics.

[9] Wolf J.B., Ferguson-Smith A.C., Lorenz A. (2022). Mendel’s laws of heredity on his 200th birthday: What have we learned by considering exceptions?. Heredity.

[10] Jessop A. (2022). Mendel in and after His Time. http://philsci-archive.pitt.edu/20332/.

[11] Chung K.P., Gonzalez-Duran E., Ruf S., Endries P., Bock R. (2023). Control of plastid inheritance by environmental and genetic factors. Nat. Plants.

[12] Yoosefzadeh-Najafabadi M., Rajcan I., Vazin M. (2022). High-throughput plant breeding approaches: Moving along with plant-based food demands for pet food industries. Front. Vet. Sci..

[13] Yoosefzadeh Najafabadi M., Hesami M., Eskandari M. (2023). Machine Learning-Assisted Approaches in Modernized Plant Breeding Programs. Genes.

[14] Yoosefzadeh Najafabadi M., Soltani F., Noory H., Díaz-Pérez J.C. (2018). Growth, yield and enzyme activity response of watermelon accessions exposed to irrigation water déficit. Int. J. Veg. Sci..

[15] Thakur R.K., Prasad P., Bhardwaj S., Gangwar O., Kumar S. (2022). Epigenetics of wheat–rust interaction: An update. Planta.

[16] Chen C., Wang M., Zhu J., Tang Y., Zhang H., Zhao Q., Jing M., Chen Y., Xu X., Jiang J. (2022). Long-term effect of epigenetic modification in plant–microbe interactions: Modification of DNA methylation induced by plant growth-promoting bacteria mediates promotion process. Microbiome.

[17] Graudal L., Dawson I.K., Hale I., Powell W., Hendre P., Jamnadass R. (2022). ‘Systems approach’plant breeding illustrated by trees. Trends Plant Sci..

[18] Bowerman A.F., Byrt C.S., Roy S.J., Whitney S.M., Mortimer J.C., Ankeny R.A., Gilliham M., Zhang D., Millar A.A., Rebetzke G.J. (2023). Potential abiotic stress targets for modern genetic manipulation. Plant Cell.

[19] Burson B.L., Young B.A. (2000). Breeding and improvement of tropical grasses. Tropical Forage Plants: Development and Use.

[20] Ranney T.G. Polyploidy: From Evolution to New Plant Development. https://ena.ipps.org/uploads/docs/56_85.pdf.

[21] Linder C.R., Rieseberg L.H. (2004). Reconstructing patterns of reticulate evolution in plants. Am. J. Bot..

[22] Schoen D.J., Lloyd D.G. (1992). Self-and cross-fertilization in plants. III. Methods for studying modes and functional aspects of self-fertilization. Int. J. Plant Sci..

[23] Bradshaw J.E. (2022). Breeding Diploid F1 Hybrid Potatoes for Propagation from Botanical Seed (TPS): Comparisons with Theory and Other Crops. Plants.

[24] De Meeûs T., Prugnolle F., Agnew P. (2007). Asexual reproduction: Genetics and evolutionary aspects. Cell. Mol. Life Sci..

[25] Cornaro L., Banfi C., Cucinotta M., Colombo L., van Dijk P.J. (2023). Asexual Reproduction through Seeds: The Complex Case of Diplosporous Apomixis.

[26] Yoosefzadeh-Najafabadi M., Rajcan I., Eskandari M. (2022). Optimizing genomic selection in soybean: An important improvement in agricultural genomics. Heliyon.

[27] van Rengs W.M., Schmidt M.H.W., Effgen S., Le D.B., Wang Y., Zaidan M.W.A.M., Huettel B., Schouten H.J., Usadel B., Underwood C.J. (2022). A chromosome scale tomato genome built from complementary PacBio and Nanopore sequences alone reveals extensive linkage drag during breeding. Plant J..

[28] Ellis T.N., Hofer J.M., Timmerman-Vaughan G.M., Coyne C.J., Hellens R.P. (2011). Mendel, 150 years on. Trends Plant Sci..

[29] Blixt S. (1975). Why didn’t Gregor Mendel find linkage?. Nature.

[30] Mittelsten Scheid O. (2022). Mendelian and non-Mendelian genetics in model plants. Plant Cell.

[31] Barker M.S., Arrigo N., Baniaga A.E., Li Z., Levin D.A. (2016). On the relative abundance of autopolyploids and allopolyploids. New Phytol..

[32] Soltis D.E., Soltis P.S., Schemske D.W., Hancock J.F., Thompson J.N., Husband B.C., Judd W.S. (2007). Autopolyploidy in angiosperms: Have we grossly underestimated the number of species?. Taxon.

[33] Soltis D.E., Buggs R.J., Doyle J.J., Soltis P.S. (2010). What we still don’t know about polyploidy. Taxon.

[34] Scott A.D., Van de Velde J.D., Novikova P.Y. (2023). Inference of polyploid origin and inheritance mode from population genomic data. Polyploidy: Methods and Protocols.

[35] Osborn T.C., Pires J.C., Birchler J.A., Auger D.L., Chen Z.J., Lee H.-S., Comai L., Madlung A., Doerge R., Colot V. (2003). Understanding mechanisms of novel gene expression in polyploids. Trends Genet..

[36] Levin D.A. (2002). The Role of Chromosomal Change in Plant Evolution.

[37] Crow J.F. (1994). Hitoshi Kihara, Japan’s pioneer geneticist. Genetics.

[38] Comai L. (2005). The advantages and disadvantages of being polyploid. Nat. Rev. Genet..

[39] Levings III C., Alexander D. (1966). Double reduction in autotetraploid maize. Genetics.

[40] Te Beest M., Le Roux J.J., Richardson D.M., Brysting A.K., Suda J., Kubešová M., Pyšek P. (2012). The more the better? The role of polyploidy in facilitating plant invasions. Ann. Bot..

[41] Gallais A. (2003). Quantitative Genetics and Breeding Methods in Autopolyploid Plants.

[42] Orr B., Godek K.M., Compton D. (2015). Aneuploidy. Curr. Biol..

[43] Zhu J., Tsai H.-J., Gordon M.R., Li R. (2018). Cellular stress associated with aneuploidy. Dev. Cell.

[44] Worland A., Gale M., Law C. (1987). Wheat genetics. Wheat Breeding: Its Scientific Basis.

[45] Hobza R., Hudzieczek V., Kubat Z., Cegan R., Vyskot B., Kejnovsky E., Janousek B. (2018). Sex and the flower–developmental aspects of sex chromosome evolution. Ann. Bot..

[46] Banaei-Moghaddam A.M., Martis M.M., Macas J., Gundlach H., Himmelbach A., Altschmied L., Mayer K.F., Houben A. (2015). Genes on B chromosomes: Old questions revisited with new tools. Biochim. Biophys. Acta (BBA)-Gene Regul. Mech..

[47] Birchler J.A., Veitia R.A. (2007). The gene balance hypothesis: From classical genetics to modern genomics. Plant Cell.

[48] Jones N., Ruban A. (2019). Are B chromosomes useful for crop improvement?. Plants People Planet.

[49] Berdnikov V., Gorel F., Kosterin O., Bogdanova V. (2003). Tertiary trisomics in the garden pea as a model of B chromosome evolution in plants. Heredity.

[50] Camus M.F., Alexander-Lawrie B., Sharbrough J., Hurst G.D. (2022). Inheritance through the cytoplasm. Heredity.

[51] Kowles R., Kowles R. (2001). Non-Mendelian Inheritance. Solving Problems in Genetics.

[52] Vinod K. (2005). Cytoplasmic genetic male sterility in plants. A molecular perspective. Proceedings of the Training Programme on Advances and Accomplishments in Heteron Breeding.

[53] Toriyama K. (2021). Molecular basis of cytoplasmic male sterility and fertility restoration in rice. Plant Biotechnol..

[54] Melonek J., Duarte J., Martin J., Beuf L., Murigneux A., Varenne P., Comadran J., Specel S., Levadoux S., Bernath-Levin K. (2021). The genetic basis of cytoplasmic male sterility and fertility restoration in wheat. Nat. Commun..

[55] Xu F., Yang X., Zhao N., Hu Z., Mackenzie S.A., Zhang M., Yang J. (2022). Exploiting sterility and fertility variation in cytoplasmic male sterile vegetable crops. Hortic. Res..

[56] Morales F., Ancín M., Fakhet D., González-Torralba J., Gámez A.L., Seminario A., Soba D., Ben Mariem S., Garriga M., Aranjuelo I. (2020). Photosynthetic metabolism under stressful growth conditions as a bases for crop breeding and yield improvement. Plants.

[57] Gualberto J.M., Mileshina D., Wallet C., Niazi A.K., Weber-Lotfi F., Dietrich A. (2014). The plant mitochondrial genome: Dynamics and maintenance. Biochimie.

[58] Forner J., Kleinschmidt D., Meyer E.H., Fischer A., Morbitzer R., Lahaye T., Schöttler M.A., Bock R. (2022). Targeted introduction of heritable point mutations into the plant mitochondrial genome. Nat. Plants.

[59] Rauf S. (2019). Breeding strategies for sunflower (*Helianthus annuus* L.) genetic improvement. Adv. Plant Breed. Strateg. Ind. Food Crops.

[60] Luo D., Xu H., Liu Z., Guo J., Li H., Chen L., Fang C., Zhang Q., Bai M., Yao N. (2013). A detrimental mitochondrial-nuclear interaction causes cytoplasmic male sterility in rice. Nat. Genet..

[61] Hanson M.R., Bentolila S. (2004). Interactions of mitochondrial and nuclear genes that affect male gametophyte development. Plant Cell.

[62] Park H.-S., Lee W.K., Lee S.-C., Lee H.O., Joh H.J., Park J.Y., Kim S., Song K., Yang T.-J. (2021). Inheritance of chloroplast and mitochondrial genomes in cucumber revealed by four reciprocal F1 hybrid combinations. Sci. Rep..

[63] Heinke L. (2023). Chilling paternal chloroplasts. Nat. Rev. Mol. Cell Biol..

[64] Dobrogojski J., Adamiec M., Luciński R. (2020). The chloroplast genome: A review. Acta Physiol. Plant..

[65] Henry R.J., Rice N., Edwards M., Nock C.J. (2014). Next-generation technologies to determine plastid genome sequences. Chloroplast Biotechnol. Methods Protoc..

[66] Nock C.J., Waters D.L., Edwards M.A., Bowen S.G., Rice N., Cordeiro G.M., Henry R.J. (2011). Chloroplast genome sequences from total DNA for plant identification. Plant Biotechnol. J..

[67] Ananda G., Norton S., Blomstedt C., Furtado A., Møller B., Gleadow R., Henry R. (2021). Phylogenetic relationships in the Sorghum genus based on sequencing of the chloroplast and nuclear genes. Plant Genome.

[68] Brozynska M., Copetti D., Furtado A., Wing R.A., Crayn D., Fox G., Ishikawa R., Henry R.J. (2017). Sequencing of Australian wild rice genomes reveals ancestral relationships with domesticated rice. Plant Biotechnol. J..

[69] Healey A., Lee D.J., Furtado A., Henry R.J. (2018). Evidence of inter-sectional chloroplast capture in Corymbia among sections Torellianae and Maculatae. Aust. J. Bot..

[70] Yue Y., Li J., Sun X., Li Z., Jiang B. (2023). Polymorphism analysis of the chloroplast and mitochondrial genomes in soybean. BMC Plant Biol..

[71] Rönspies M., Schindele P., Puchta H. (2021). CRISPR/Cas-mediated chromosome engineering: Opening up a new avenue for plant breeding. J. Exp. Bot..

[72] Prieto P. (2020). Chromosome manipulation for plant breeding purposes. Agronomy.

[73] Badaeva E., Dedkova O., Gay G., Pukhalskyi V., Zelenin A., Bernard S., Bernard M. (2007). Chromosomal rearrangements in wheat: Their types and distribution. Genome.

[74] Sharma S.P., Peterson T. (2023). Complex chromosomal rearrangements induced by transposons in maize. Genetics.

[75] Zhang Y., Wu Y., Li G., Qi A., Zhang Y., Zhang T., Qi Y. (2022). Genome-wide investigation of multiplexed CRISPR-Cas12a-mediated editing in rice. Plant Genome.

[76] Verlaan M.G., Szinay D., Hutton S.F., de Jong H., Kormelink R., Visser R.G., Scott J.W., Bai Y. (2011). Chromosomal rearrangements between tomato and Solanum chilense hamper mapping and breeding of the TYLCV resistance gene Ty-1. Plant J..

[77] Halfhill M.D., Warwick S.I. (2016). Mendelian genetics and plant reproduction. Plant Biotechnology and Genetics: Principles, Techniques and Applications.

[78] Mackay T.F. (2014). Epistasis and quantitative traits: Using model organisms to study gene–gene interactions. Nat. Rev. Genet..

[79] Shull G.H. (1914). Duplicate genes for capsule-form in Bursa bursa-pastoris. Z Indukt Abstamm Vererb..

[80] Yoosefzadeh-Najafabadi M., Eskandari M., Belzile F., Torkamaneh D. (2022). Genome-wide association study statistical models: A review. Methods Mol Biol..

[81] Hong H., Yoosefzadeh-Najafabadi M., Rajcan I. (2022). Correlations between soybean seed quality traits using a genome-wide association study panel grown in Canadian and Ukrainian mega-environments. Can. J. Plant Sci..

[82] Yoosefzadeh-Najafabadi M., Eskandari M., Torabi S., Torkamaneh D., Tulpan D., Rajcan I. (2022). Machine-learning-based genome-wide association studies for uncovering QTL underlying soybean yield and its components. Int. J. Mol. Sci..

[83] Carlborg Ö., Haley C.S. (2004). Epistasis: Too often neglected in complex trait studies?. Nat. Rev. Genet..

[84] Phillips P.C. (2008). Epistasis—The essential role of gene interactions in the structure and evolution of genetic systems. Nat. Rev. Genet..

[85] Hayes B.J., Lewin H.A., Goddard M.E. (2013). The future of livestock breeding: Genomic selection for efficiency, reduced emissions intensity, and adaptation. Trends Genet..

[86] Wang R., Lammers M., Tikunov Y., Bovy A.G., Angenent G.C., de Maagd R.A. (2020). The rin, nor and Cnr spontaneous mutations inhibit tomato fruit ripening in additive and epistatic manners. Plant Sci..

[87] Evans K.S., van Wijk M.H., McGrath P.T., Andersen E.C., Sterken M.G. (2021). From QTL to gene: C. elegans facilitates discoveries of the genetic mechanisms underlying natural variation. Trends Genet..

[88] Wang Q., Tang J., Han B., Huang X. (2020). Advances in genome-wide association studies of complex traits in rice. Theor. Appl. Genet..

[89] Yoosefzadeh Najafabadi M. (2021). Using Advanced Proximal Sensing and Genotyping Tools Combined with Bigdata Analysis Methods to Improve Soybean Yield. Ph.D. Thesis.

[90] Hill W.G., Goddard M.E., Visscher P.M. (2008). Data and theory point to mainly additive genetic variance for complex traits. PLoS Genet..

[91] Deutschbauer A.M., Davis R.W. (2005). Quantitative trait loci mapped to single-nucleotide resolution in yeast. Nat. Genet..

[92] Gerke J., Lorenz K., Cohen B. (2009). Genetic interactions between transcription factors cause natural variation in yeast. Science.

[93] Rowe H.C., Hansen B.G., Halkier B.A., Kliebenstein D.J. (2008). Biochemical networks and epistasis shape the Arabidopsis thaliana metabolome. Plant Cell.

[94] Doebley J., Stec A., Gustus C. (1995). teosinte branched1 and the origin of maize: Evidence for epistasis and the evolution of dominance. Genetics.

[95] Kroymann J., Mitchell-Olds T. (2005). Epistasis and balanced polymorphism influencing complex trait variation. Nature.

[96] Mackay T.F. (2015). Epistasis for quantitative traits in Drosophila. Epistasis Methods Protoc..

[97] Shao H., Burrage L.C., Sinasac D.S., Hill A.E., Ernest S.R., O’Brien W., Courtland H.-W., Jepsen K.J., Kirby A., Kulbokas E. (2008). Genetic architecture of complex traits: Large phenotypic effects and pervasive epistasis. Proc. Natl. Acad. Sci. USA.

[98] Greene C.S., Penrod N.M., Williams S.M., Moore J.H. (2009). Failure to replicate a genetic association may provide important clues about genetic architecture. PLoS ONE.

[99] Waddington C.H. (1942). Canalization of development and the inheritance of acquired characters. Nature.

[100] Yamamoto A., Anholt R.R., Mackay T.F. (2009). Epistatic interactions attenuate mutations affecting startle behaviour in Drosophila melanogaster. Genet. Res..

[101] Rutherford S.L., Lindquist S. (1998). Hsp90 as a capacitor for morphological evolution. Nature.

[102] Sangster T.A., Salathia N., Lee H.N., Watanabe E., Schellenberg K., Morneau K., Wang H., Undurraga S., Queitsch C., Lindquist S. (2008). HSP90-buffered genetic variation is common in Arabidopsis thaliana. Proc. Natl. Acad. Sci. USA.

[103] Gallusci P., Agius D.R., Moschou P.N., Dobránszki J., Kaiserli E., Martinelli F. (2023). Deep inside the epigenetic memories of stressed plants. Trends Plant Sci..

[104] Ramakrishnan M., Papolu P.K., Satish L., Vinod K.K., Wei Q., Sharma A., Emamverdian A., Zou L.-H., Zhou M. (2022). Redox status of the plant cell determines epigenetic modifications under abiotic stress conditions and during developmental processes. J. Adv. Res..

[105] Sobral M., Sampedro L. (2022). Phenotypic, epigenetic, and fitness diversity within plant genotypes. Trends Plant Sci..

[106] Hesami M., Jones A.M.P. (2023). Potential roles of epigenetic memory on the quality of clonal cannabis plants: Content and profile of secondary metabolites. Med. Usage Cannabis Cannabinoids.

[107] Lloyd J.P.B., Lister R. (2022). Epigenome plasticity in plants. Nat. Rev. Genet..

[108] Kalisz S., Purugganan M.D. (2004). Epialleles via DNA methylation: Consequences for plant evolution. Trends Ecol. Evol..

[109] Zhang Y., Wendte J.M., Ji L., Schmitz R.J. (2020). Natural variation in DNA methylation homeostasis and the emergence of epialleles. Proc. Natl. Acad. Sci. USA.

[110] Kakutani T. (2002). Epi-Alleles in Plants: Inheritance of Epigenetic Information over Generations. Plant Cell Physiol..

[111] Weigel D., Colot V. (2012). Epialleles in plant evolution. Genome Biol..

[112] House M., Lukens L., Alvarez-Venegas R., De-la-Peña C., Casas-Mollano J.A. (2019). The Role of Germinally Inherited Epialleles in Plant Breeding: An Update. Epigenetics in Plants of Agronomic Importance: Fundamentals and Applications: Transcriptional Regulation and Chromatin Remodelling in Plants.

[113] Hudzieczek V., Hobza R., Cápal P., Šafář J., Doležel J. (2022). If Mendel Was Using CRISPR: Genome Editing Meets Non-Mendelian Inheritance. Adv. Funct. Mater..

[114] Casas E., Vavouri T. (2020). Mechanisms of epigenetic inheritance of variable traits through the germline. Reproduction.

[115] Srikant T., Tri Wibowo A. (2021). The Underlying Nature of Epigenetic Variation: Origin, Establishment, and Regulatory Function of Plant Epialleles. Int. J. Mol. Sci..

[116] Quadrana L., Etcheverry M., Gilly A., Caillieux E., Madoui M.-A., Guy J., Bortolini Silveira A., Engelen S., Baillet V., Wincker P. (2019). Transposition favors the generation of large effect mutations that may facilitate rapid adaption. Nat. Commun..

[117] Quadrana L., Almeida J., Asís R., Duffy T., Dominguez P.G., Bermúdez L., Conti G., Corrêa da Silva J.V., Peralta I.E., Colot V. (2014). Natural occurring epialleles determine vitamin E accumulation in tomato fruits. Nat. Commun..

[118] Manning K., Tör M., Poole M., Hong Y., Thompson A.J., King G.J., Giovannoni J.J., Seymour G.B. (2006). A naturally occurring epigenetic mutation in a gene encoding an SBP-box transcription factor inhibits tomato fruit ripening. Nat. Genet..

[119] Martin A., Troadec C., Boualem A., Rajab M., Fernandez R., Morin H., Pitrat M., Dogimont C., Bendahmane A. (2009). A transposon-induced epigenetic change leads to sex determination in melon. Nature.

[120] Wei X., Song X., Wei L., Tang S., Sun J., Hu P., Cao X. (2017). An epiallele of rice AK1 affects photosynthetic capacity. J. Integr. Plant Biol..

[121] Eichten S.R., Briskine R., Song J., Li Q., Swanson-Wagner R., Hermanson P.J., Waters A.J., Starr E., West P.T., Tiffin P. (2013). Epigenetic and Genetic Influences on DNA Methylation Variation in Maize Populations. Plant Cell.

[122] Eichten S.R., Swanson-Wagner R.A., Schnable J.C., Waters A.J., Hermanson P.J., Liu S., Yeh C.-T., Jia Y., Gendler K., Freeling M. (2011). Heritable Epigenetic Variation among Maize Inbreds. PLOS Genet..

[123] Li Q., Eichten S.R., Hermanson P.J., Springer N.M. (2014). Inheritance Patterns and Stability of DNA Methylation Variation in Maize Near-Isogenic Lines. Genetics.

[124] Zhang Y.-Y., Fischer M., Colot V., Bossdorf O. (2013). Epigenetic variation creates potential for evolution of plant phenotypic plasticity. New Phytol..

[125] Miura K., Agetsuma M., Kitano H., Yoshimura A., Matsuoka M., Jacobsen S.E., Ashikari M. (2009). A metastable DWARF1 epigenetic mutant affecting plant stature in rice. Proc. Natl. Acad. Sci. USA.

[126] Xu J., Chen G., Hermanson P.J., Xu Q., Sun C., Chen W., Kan Q., Li M., Crisp P.A., Yan J. (2019). Population-level analysis reveals the widespread occurrence and phenotypic consequence of DNA methylation variation not tagged by genetic variation in maize. Genome Biol..

[127] Yoosefzadeh Najafabadi M., Rajcan I. (2022). Six Decades of Soybean Breeding in Ontario, Canada: A Tradition of Innovation. Can. J. Plant Sci..

[128] Stoltenberg S.F. (2022). Foundations of Behavior Genetics.

[129] Darden L. (1980). Theory construction in genetics. Scientific Discovery: Case Studies.

[130] Sinha S., Kushwaha B.K., Deshmukh R.K. (2022). QTL Mapping Using Advanced Mapping Populations and High-throughput Genotyping. Genotyping by Sequencing for Crop Improvement.

[131] Rani K., Kumar M., Razzaq A., Ajay B., Kona P., Bera S.K., Wani S.H. (2023). Recent advances in molecular marker technology for QTL mapping in plants. QTL Mapping in Crop Improvement.

[132] Guo B., Wang D., Guo Z., Beavis W.D. (2013). Family-based association mapping in crop species. Theor. Appl. Genet..

[133] Darvasi A., Weinreb A., Minke V., Weller J., Soller M. (1993). Detecting marker-QTL linkage and estimating QTL gene effect and map location using a saturated genetic map. Genetics.

[134] Andersson L., Georges M. (2004). Domestic-animal genomics: Deciphering the genetics of complex traits. Nat. Rev. Genet..

[135] Yu J., Pressoir G., Briggs W.H., Vroh Bi I., Yamasaki M., Doebley J.F., McMullen M.D., Gaut B.S., Nielsen D.M., Holland J.B. (2006). A unified mixed-model method for association mapping that accounts for multiple levels of relatedness. Nat. Genet..

[136] Kingsmore S.F., Lindquist I.E., Mudge J., Gessler D.D., Beavis W.D. (2008). Genome-wide association studies: Progress and potential for drug discovery and development. Nat. Rev. Drug Discov..

[137] Buckler E.S., Holland J.B., Bradbury P.J., Acharya C.B., Brown P.J., Browne C., Ersoz E., Flint-Garcia S., Garcia A., Glaubitz J.C. (2009). The genetic architecture of maize flowering time. Science.

[138] Guo B., Sleper D., Sun J., Nguyen H., Arelli P., Shannon J. (2006). Pooled analysis of data from multiple quantitative trait locus mapping populations. Theor. Appl. Genet..

[139] Tian F., Bradbury P., Brown P., Sun Q., Flint-Garcia S., Rocheford T., McMullen M., Holland J., Buckler E. (2011). Genome-wide association study of maize identifies genes affecting leaf architecture. Nat. Genet..

[140] Laird N.M., Lange C. (2006). Family-based designs in the age of large-scale gene-association studies. Nat. Rev. Genet..

[141] Jansen R.C., Jannink J.L., Beavis W.D. (2003). Mapping quantitative trait loci in plant breeding populations: Use of parental haplotype sharing. Crop Sci..

[142] Xu S. (2003). Theoretical basis of the Beavis effect. Genetics.

[143] Holland J.B. (2007). Genetic architecture of complex traits in plants. Curr. Opin. Plant Biol..

[144] Beavis W.D. (2019). QTL analyses: Power, precision, and accuracy. Molecular Dissection of Complex Traits.

[145] Beavis W., Wilkinson D. (1994). Proceedings of the Forty-Ninth Annual Corn and Sorghum Industry Research Conference.

[146] Paterson A.H., Press C. (1998). Molecular Dissection of Complex Traits.

[147] Otto S.P., Jones C.D. (2000). Detecting the undetected: Estimating the total number of loci underlying a quantitative trait. Genetics.

[148] Hayes B., Goddard M.E. (2001). The distribution of the effects of genes affecting quantitative traits in livestock. Genet. Sel. Evol..

[149] Blackburn A., Sidhu G., Schillinger W.F., Skinner D., Gill K. (2021). QTL mapping using GBS and SSR genotyping reveals genomic regions controlling wheat coleoptile length and seedling emergence. Euphytica.

[150] Hall D., Hallingbäck H.R., Wu H.X. (2016). Estimation of number and size of QTL effects in forest tree traits. Tree Genet. Genomes.

[151] Wellenreuther M., Hansson B. (2016). Detecting polygenic evolution: Problems, pitfalls, and promises. Trends Genet..

[152] King E.G., Long A.D. (2017). The Beavis effect in next-generation mapping panels in Drosophila melanogaster. G3 Genes Genomes Genet..

[153] Heil C.S.S., DeSevo C.G., Pai D.A., Tucker C.M., Hoang M.L., Dunham M.J. (2017). Loss of heterozygosity drives adaptation in hybrid yeast. Mol. Biol. Evol..

[154] Tutaj H., Pirog A., Tomala K., Korona R. (2022). Genome-scale patterns in the loss of heterozygosity incidence in Saccharomyces cerevisiae. Genetics.

[155] Wang R.R.-C., Li X.-M., Chatterton N. (1999). Loss of heterozygosity and accelerated genotype fixation in rice hybrids. Genome.

[156] Wang R.R.-C., Li X.-M., Chatterton N.J. (2001). A proposed mechanism for loss of heterozygosity in rice hybrids. Euphytica.

[157] Wang R.R.-C., Li X.-M., Chatterton N.J. (2006). Cytological evidence for assortment mitosis leading to loss of heterozygosity in rice. Genome.

